# Supplementary material for: 8-Hydroxydaidzein, an Isoflavone from Fermented Soybean, Induces Autophagy, Apoptosis, Differentiation, and Degradation of Oncoprotein BCR-ABL in K562 Cells
Source: Biomedicines. 2020 Nov 16;8(11):506. doi: 10.3390/biomedicines8110506 (PMC7696406; doi:10.3390/biomedicines8110506)
Supplement: Supplementary file 1 [file biomedicines-08-00506-s001.pdf]

Supplemental Materials

Table S1. The most significant regulated genes by 8-OHD (100 μM) in K562 cells.

ID	Symbol	log_2FC	*p* Value	*q* Value
4017	*LOXL2*	7.54	2.26E-05	5.02E-03
2051	*EPHB6*	5.16	7.95E-05	6.12E-03
885	*CCK*	4.89	3.77E-06	3.78E-03
6281	*S100A10*	4.89	1.76E-04	7.72E-03
3866	*KRT15*	4.88	2.63E-05	5.26E-03
1294	*COL7A1*	4.83	3.83E-04	9.43E-03
2669	*GEM*	4.58	1.11E-04	6.82E-03
1191	*CLU*	4.45	6.57E-07	2.77E-03
221476	*PI16*	4.45	1.47E-06	2.77E-03
5054	*SERPINE1*	4.38	2.40E-05	5.15E-03
9610	*RIN1*	4.31	3.48E-05	5.32E-03
3566	*IL4R*	4.31	4.98E-04	1.06E-02
3604	*TNFRSF9*	4.15	3.24E-04	9.02E-03
10381	*TUBB3*	4.12	3.92E-05	5.34E-03
5329	*PLAUR*	4.11	9.85E-04	1.32E-02
969	*CD69*	-3.82	1.02E-04	6.56E-03
6941	*TCF19*	-3.85	1.40E-05	4.50E-03
492307	*C8orf22*	-3.91	6.81E-04	1.15E-02
55274	*PHF10*	-4.03	2.10E-04	7.99E-03
7923	*HSD17B8*	-4.07	1.39E-03	1.51E-02
6296	*ACSM3*	-4.07	5.43E-04	1.08E-02
138311	*FAM69B*	-4.10	1.85E-04	7.80E-03
55808	*ST6GALNAC1*	-4.21	1.41E-04	7.26E-03
462	*SERPINC1*	-4.26	1.87E-04	7.80E-03
159989	*CCDC67*	-4.47	2.29E-04	8.02E-03
1572	*CYP2F1*	-4.65	3.50E-05	5.32E-03
445	*ASS1*	-4.73	4.02E-06	3.78E-03
138255	*C9orf135*	-4.73	7.93E-04	1.21E-02
83729	*INHBE*	-4.94	1.18E-05	4.35E-03
57520	*HECW2*	-6.01	8.33E-04	1.22E-02

Table S2. Overrepresented gene sets in DAVID GO BP DIRECT annotations database obtained from comparisons in gene expression profiles between 100 μM 8-OHD treatment vs. vehicle control ($p < 0.05$ and minimal number of genes per group >=30).

Term	Count	%	*p* value
GO:0007264~small GTPase mediated signal transduction	58	1.83	2.81E-03
**GO:0006914~autophagy**	**35**	**1.10**	**3.81E-03**
GO:0006366~transcription from RNA polymerase II promoter	107	3.37	4.26E-03
GO:0045944~positive regulation of transcription from RNA polymerase II promoter	189	5.95	6.43E-03
GO:0006468~protein phosphorylation	95	2.99	7.27E-03
GO:0006357~regulation of transcription from RNA polymerase II promoter	92	2.90	7.94E-03
GO:0007568~aging	40	1.26	8.53E-03
GO:0008360~regulation of cell shape	35	1.10	8.83E-03
GO:0045893~positive regulation of transcription, DNA-templated	105	3.31	9.12E-03
GO:0006461~protein complex assembly	30	0.95	9.87E-03
GO:0042127~regulation of cell proliferation	43	1.35	1.32E-02
GO:0006886~intracellular protein transport	52	1.64	1.77E-02
GO:0051607~defense response to virus	38	1.20	2.29E-02
GO:0060271~cilium morphogenesis	32	1.01	2.84E-02
GO:0006351~transcription, DNA-templated	348	10.96	2.92E-02
GO:0006397~mRNA processing	40	1.26	3.06E-02
GO:0051092~positive regulation of NF-kappaB transcription factor activity	31	0.98	3.49E-02
GO:0042981~regulation of apoptotic process	46	1.45	3.50E-02
GO:0042493~response to drug	62	1.95	4.28E-02
GO:0008283~cell proliferation	73	2.30	4.42E-02
GO:0001525~angiogenesis	47	1.48	4.78E-02

Table S3. The KEGG pathways associated with 100 μM 8-OHD treatment in K562 cells

KEGG Pathway	gene count	%	*p* Value
hsa01100:Metabolic pathways	261	8.223	4.82E-07
hsa00760:Nicotinate and nicotinamide metabolism	15	0.473	6.55E-05
hsa04142:Lysosome	37	1.166	1.67E-04
hsa03018:RNA degradation	24	0.756	2.27E-03
hsa05231:Choline metabolism in cancer	29	0.914	2.77E-03
hsa04146:Peroxisome	25	0.788	2.97E-03
hsa05202:Transcriptional misregulation in cancer	42	1.323	4.20E-03
hsa01230:Biosynthesis of amino acids	22	0.693	4.72E-03
hsa05211:Renal cell carcinoma	20	0.630	8.16E-03
hsa01200:Carbon metabolism	29	0.914	1.43E-02
hsa01130:Biosynthesis of antibiotics	48	1.512	1.67E-02
hsa04950:Maturity onset diabetes of the young	10	0.315	1.87E-02
hsa05120:Epithelial cell signaling in Helicobacter pylori infection	19	0.599	2.03E-02
hsa04010:MAPK signaling pathway	55	1.733	2.24E-02
hsa04966:Collecting duct acid secretion	10	0.315	2.41E-02
hsa04152:AMPK signaling pathway	30	0.945	2.48E-02
hsa04662:B cell receptor signaling pathway	19	0.599	2.71E-02
hsa04666:Fc gamma R-mediated phagocytosis	22	0.693	2.83E-02
hsa00600:Sphingolipid metabolism	14	0.441	3.58E-02
hsa05323:Rheumatoid arthritis	22	0.693	4.52E-02
hsa05166:HTLV-I infection	53	1.670	4.93E-02

Table S4. The BioCarta pathways associated with 100 μM 8-OHD treatment in K562 cells

BioCarta pathway	gene count	%	*p* Value
Signaling Pathway from G-Protein Families	15	0.473	1.07E-03
Signaling of Hepatocyte Growth Factor Receptor	15	0.473	2.07E-03
NFAT and Hypertrophy of the heart (Transcription in the broken heart)	19	0.599	3.77E-03
Fc Epsilon Receptor I Signaling in Mast Cells	15	0.473	4.91E-03
TPO Signaling Pathway	11	0.347	5.09E-03
PDGF Signaling Pathway	12	0.378	5.69E-03
Mechanism of Gene Regulation by Peroxisome Proliferators via PPARa(alpha)	18	0.567	6.06E-03
IGF-1 Signaling Pathway	10	0.315	6.28E-03
T Cell Receptor Signaling Pathway	16	0.504	6.34E-03
MAPKinase Signaling Pathway	26	0.819	6.61E-03
The IGF-1 Receptor and Longevity	8	0.252	9.19E-03
BCR Signaling Pathway	13	0.410	1.05E-02
Effects of calcineurin in Keratinocyte Differentiation	9	0.284	1.12E-02
Inhibition of Cellular Proliferation by Gleevec	10	0.315	1.25E-02
EGF Signaling Pathway	11	0.347	1.33E-02
IL-2 Receptor Beta Chain in T cell Activation	14	0.441	1.33E-02
Insulin Signaling Pathway	9	0.284	2.18E-02
Control of skeletal myogenesis by HDAC & calcium/calmodulin-dependent kinase (CaMK)	11	0.347	2.26E-02
METS affect on Macrophage Differentiation	8	0.252	2.80E-02
Role of Erk5 in Neuronal Survival	8	0.252	2.80E-02
Regulation of BAD phosphorylation	10	0.315	2.93E-02
B Cell Survival Pathway	7	0.221	3.60E-02
Corticosteroids and cardioprotection	8	0.252	3.78E-02
CXCR4 Signaling Pathway	9	0.284	4.85E-02
Nerve growth factor pathway (NGF)	8	0.252	4.96E-02

Neurogenesis_NGF TrkA MAPK-mediated signaling

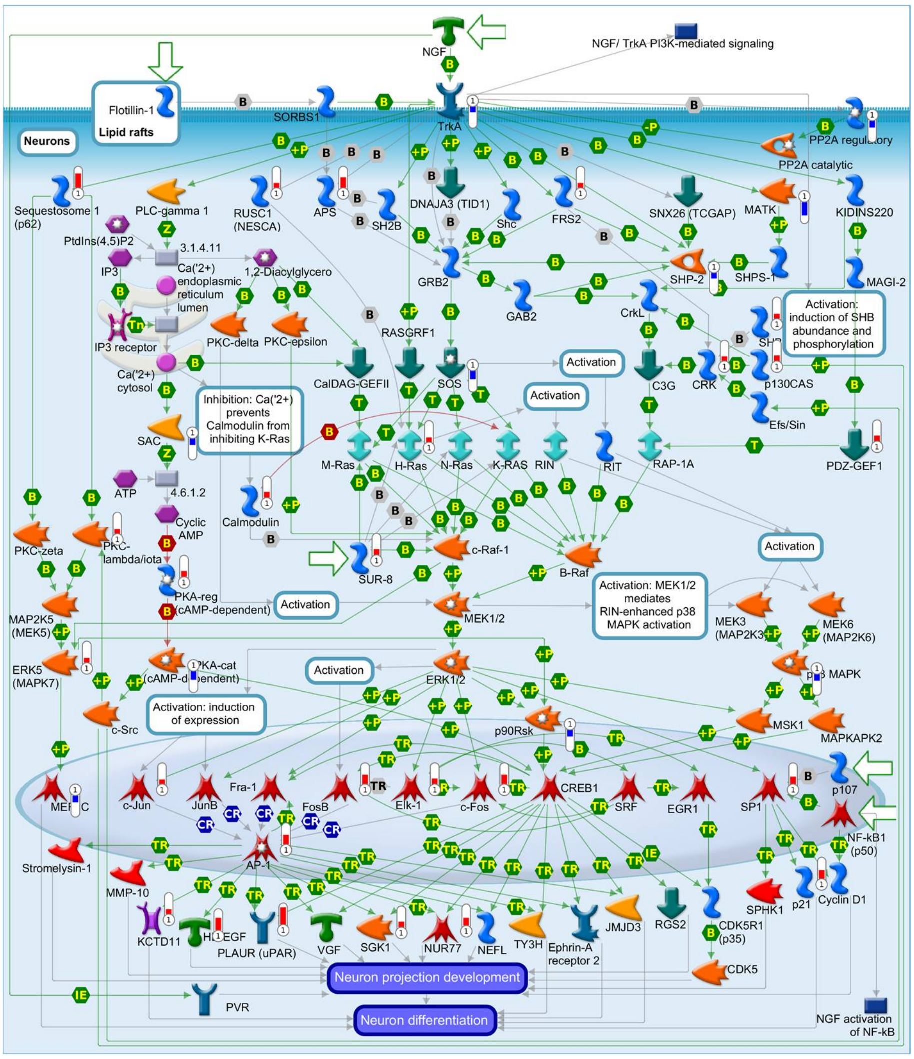

Figure S1. MetaCore canonical pathway map showing neurogenesis NGF/TrkA MAPK signaling pathway in response to 8-OHD. DEGs are denoted with thermometer-like icons (red=upregulation, blue =downregulation).

Apoptosis_Anti-Apoptosis mediated by external signals via MAPK and JAK_STAT

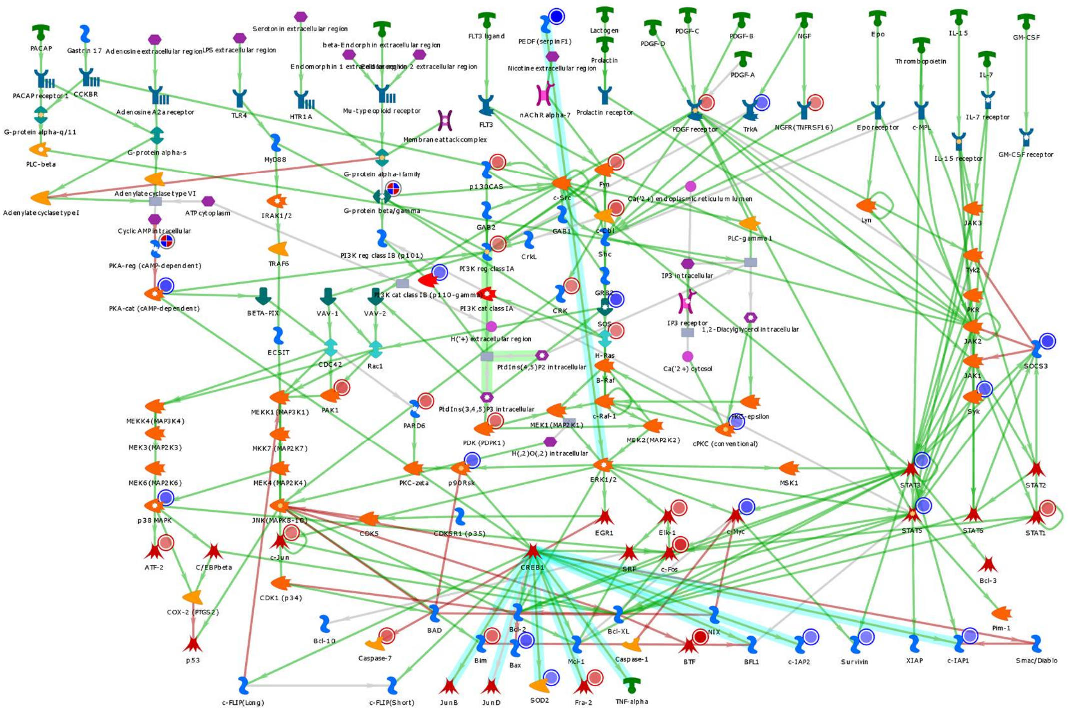

Figure S2. MetaCore process network map showing apoptosis/anti-apoptosis mediated by external signals via MAPK and JAK/STAT in response to 8-OHD. DEGs are denoted with a circle (red=upregulation, blue =downregulation).
